# Barriers in Bangladesh

**DOI:** 10.7554/eLife.41926

**Published:** 2018-09-20

**Authors:** Senjuti Saha, Sudipta Saha, Samir K Saha

**Affiliations:** 1 Child Health Research Foundation, Department of MicrobiologyDhaka Shishu HospitalDhakaBangladesh; 2 Department of Infectious DiseasesStanford University School of MedicineStanfordUnited States; 3 Department of Global Health and PopulationHarvard T.H. Chan School of Public HealthBostonUnited States; 4 Bangladesh Institute of Child HealthDhaka Shishu HospitalDhakaBangladesh

**Keywords:** global science, science policy, careers in science, diversity, scientific excellence, productivity

## Abstract

Research laboratories in low- and middle-income countries, where the global burden of disease is highest, face systemic challenges in conducting research and public health surveillance. An international effort is needed to overcome the paywalls, customs regulations and lack of local suppliers that hinder the scientific community in these countries.

In September 1995, two teams from the United States arrived at Dhaka Shishu (Children’s) Hospital in Bangladesh to evaluate and improve guidelines for referring sick children to hospitals (Kalter et al., 1997). The protocol required measuring the haemoglobin concentrations in children’s blood samples – a straightforward task performed using a device called a HemoCue. However, although it had been ordered before the scientists arrived, the HemoCue was stranded at customs clearance in Bangladesh. Three months lapsed before it arrived in our laboratory and the project could begin.

We took a picture to celebrate the moment the HemoCue arrived (Figure 1), and it still hangs on the fridge of our laboratory. The glowing faces reflect how a simple machine became invaluable and extraordinarily expensive, costing us three months of work and personnel time.

**Figure 1.**
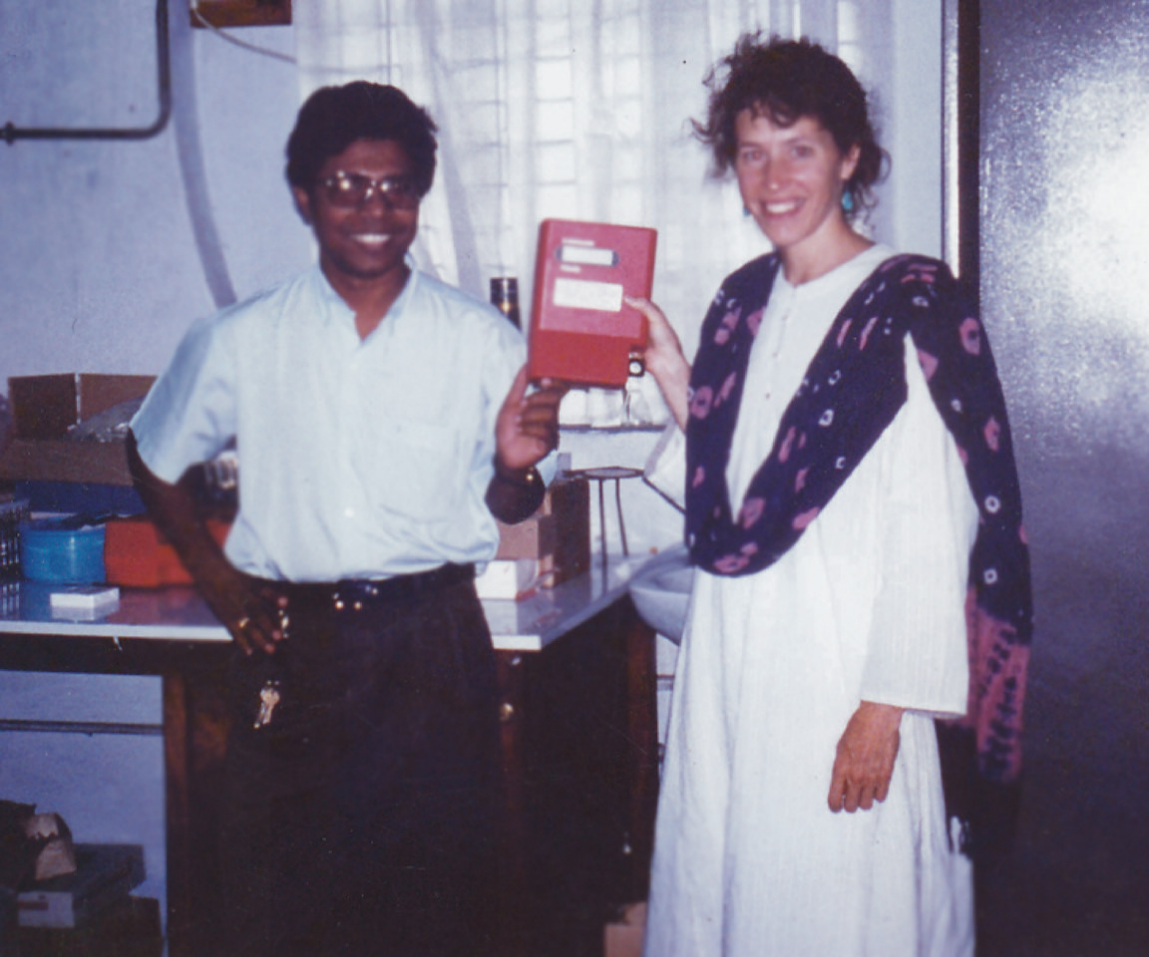
Samir Saha (left) of Dhaka Shishu Hospital (DSH) in Bangladesh and Julie Schillinger of the Centers for Disease Control and Prevention in the United States pose with the HemoCue in the DSH microbiology laboratory. Problems getting the HemoCue through customs delayed the start of a public health project by three months; such delays are just one of the challenges that researchers in Bangladesh must contend with.

IMAGE CREDIT: Maksuda Islam, Child Health Research Foundation.

## Wasted time, blunted motivation

Research laboratories form the backbone of public health surveillance systems. In the last two decades, with support from groups like the Wellcome Trust, the Bill and Melinda Gates Foundation (BMGF), and the World Health Organization (WHO), low- and middle-income countries (LMICs) have experienced an enormous increase in research funding. However, the systemic and daily hindrances to conducting research and obtaining laboratory supplies and equipment have not improved proportionately. According to the WHO, the scarcity of health technologies in LMICs was partly responsible for the slow progress towards achieving the Millennium Development Goals, which aimed to reduce morbidity and mortality (World Health Organization, 2014). This, we believe, is firmly tied to the barriers that must be overcome to acquire even the most basic laboratory supplies for research.

For almost every research study we conduct in Bangladesh, we face a multitude of barriers that are almost non-existent in high-income settings. Earlier this year, three members of our team spent several weeks strategizing on how to obtain Thayer Martin media and supplements necessary to grow meningitis-causing bacteria, because domestic vendors had stopped supplying them. Following weeks of contacting different vendors and facing unreal promises of imminent delivery, we had to start contacting our friends abroad. We hoped that a colleague travelling to Bangladesh could bring some samples of the media; we could then at least start the experiments while waiting to obtain the bulk of the supplies through local sources. This is not a sustainable solution, but we needed the supplies urgently – we had already collected 1,600 specimens from children but were not able to process them. It took us over five months to secure a long-term local supply and start processing the samples.

Routine tasks like obtaining primers for polymerase chain reactions (PCRs) can be quite challenging too. For anyone working in the US or Europe, ordering primers is generally one of the easiest and fastest steps, normally taking no longer than three days for delivery. Here, however, running a PCR means thinking far ahead; the time to obtain primers can be as long as six months. There are no manufacturers within Bangladesh and clearing lab reagents through customs is still difficult. These struggles usually translate to a long lag time, often months, between conceiving an idea and performing the experiments. This, in turn, translates into blunted motivation and, often, redundant ideas. For example, we recently designed novel primers to identify mutations in antibiotic-resistant genes in *Salmonella* Typhi. By the time the primers arrived, and we finished performing the experiments, another group had published similar findings. In a scientific world where replication is undervalued it was a big loss for our team, both in terms of resources and intellectual effort. Such incidents are commonplace in most LMIC research settings but are rarely discussed or effectively acted upon.

During our journal clubs, members often talk about abstracts of exciting papers they would have presented had they not hit the paywall

## The imprisoned scholarly-poor

It can also be challenging to keep up with the literature. The current structure of academic publishing keeps results away from researchers in LMICs, while still requiring them to publish in these forums for credibility. Institutions in LMICs, specifically non-profit research organizations like ours, cannot afford to provide their members with unlimited access to scientific journals, making it impossible for these researchers to access much of the scholarly literature. During our journal clubs, members often talk about abstracts of exciting papers they would have presented had they not hit the paywall.

Over and above paywalls for access is the expense of publishing in these same journals. Recently, we completed a small project evaluating a novel tool for detecting typhoidal *Salmonella* in the environment. The overarching objective of this study was to develop a low-cost PCR-based surveillance method for LMICs that cannot afford to establish or sustain traditional blood culture-based community surveillance. The whole study, for which we received no external funding, cost $3,864 (excluding personnel costs). The journal where we submitted the manuscript estimated a cost of $1,625 for publication, with an additional $2,500 required to make it open access. Although the findings reported in the paper were specifically targeted to groups like ours in the Global South without access to most of the literature, we had no option but to renounce the cost-prohibitive open access option and, in turn, limit the potential benefits of our study. We too played into the role of keeping the scholarly poor trapped in the vicious cycle of colonial science culture.

## Global engagement towards global health

It is unacceptable that the growth of science globally is slowed down by barriers that prevent access to knowledge and research supplies. The obstacles are multifaceted, but not impossible to overcome. Examples of current efforts to improve access to the literature include the HINARI Access to Research Initiative by the WHO, which provides free or low-cost access to journals to researchers affiliated with non-profit organizations in LMICs (Research4Life, 2018). However, access to HINARI is based on GNP, so it excludes countries like India, Brazil and Indonesia, and many other countries will lose their eligibility as their economies develop (The PLoS Medicine Editors, 2006). However, a slight rise in GNP does not make research organizations in the country resource-rich overnight.

To fundamentally tackle this issue, we need global collaboration to push for universal access. Recently, several European countries and institutions have moved to cancel their subscription with some of the giant publishers due to their slow progress towards open access (Schiermeier, 2018; Yeager, 2018). Many LMICs do not individually have the platform to negotiate with publishers; however, collectively we may be able to expedite changes and shape the future of academic access. Furthermore, if countries like Sweden and Germany come together with LMICs, it could prove to be a powerful platform. Such collaborations can be extensions of the growth in research collaborations across countries.

While we wait for universal access, there are also alternative avenues for incremental progress. Many funding agencies now require all funded research to be published as open-access with associated fees being directly paid by the donor. However, it is important to not lose sight of the fact that donors usually have specific priorities, and well-intentioned efforts by individual donors can have asymmetrical impacts, making certain research topics and publications available, while contradictory findings may not be.

The idea of easy access to laboratory supplies is not far-fetched either. One solution could include donors, like the BMGF, working with local vendors or other organizations to ensure timely delivery of supplies to their grantees. This is not a fanciful idea – we were able to achieve this while conducting the ANISA (Aetiology of Neonatal Infections in South Asia) study in collaboration with the Centers for Disease Control and Prevention (CDC; Hibberd and Qazi, 2016; Saha et al., 2018). ANISA performed cutting-edge diagnostic tests in five sites, including rural sites, in India, Bangladesh and Pakistan. It was with the help of local CDC offices in these countries, who formed strong relationships with the relevant governments, that the same equipment and research supplies were delivered to all the laboratories without delays or hassles. However, recent budget cuts mean that the CDC may discontinue its work in 39 of the 49 countries it currently operates in (McKay, 2018).

Regardless of the CDC’s future in global health, the WHO has the largest role in the field by far, putting it in a crucial position to facilitate changes. As with the CDC, our experience suggests that this is feasible. The WHO runs Invasive Bacterial Vaccine Preventable Disease (IB-VPD) surveillance studies in 58 countries, where they have already set up offices that can facilitate the delivery of relevant supplies to supported laboratories (Hasan et al., 2018); the current process, however, often moves at a glacial pace and is highly bureaucratic. Donors could leverage the systems and relationships already established by the WHO to help research laboratories acquire supplies for their own projects.

More sustainably, the WHO can work with governments in LMICs to assemble reasonable customs regulations for biological research products. This would require extensive and honest partnerships and collaborations between multiple stakeholders, including donors, governments, WHO employees and local vendors, and thus extra effort beyond just monetary support in the form of research grants. The results, however, can go a long way towards improving the prospects of scientific research in LMICs.

Without immediate and sustained interventions, the gap in resources between high-resource settings and low-resource settings will only worsen, jeopardizing research in LMICs further. It is time that we all acknowledge the systemic barriers to conducting research in LMICs. By uniting as one worldwide scientific community we can take appropriate steps to bring the scholarly poor out of literary poverty and make global health research truly global.
